# Metastatic Clear Cell Renal Cell Carcinoma to Pancreas and Distant Organs 24 Years After Radical Nephrectomy: A Case Report and Literature Review

**DOI:** 10.3389/fsurg.2022.894272

**Published:** 2022-07-05

**Authors:** Huawei Cao, Zejia Sun, Jiyue Wu, Changzhen Hao, Wei Wang

**Affiliations:** ^1^ Department of Urology, Beijing Chao-yang Hospital, Capital Medical University, Beijing, China; ^2^ Institute of Urology, Capital Medical University, Beijing, China

**Keywords:** Clear cell renal cell carcinoma, pancreatic metastasis, metastasectomy, systemic therapy, combinatorial treatment

## Abstract

**Background:**

Clear cell renal cell carcinoma (CCRCC) is a common urological neoplasm, and even though surgical resection is effective for localized CCRCC, the prognosis of metastatic CCRCC is poor. Currently, there is a paucity of recognized effective therapeutic protocols for metastatic CCRCC.

**Case presentation:**

A 76-year-old Asian man underwent radical left nephrectomy for CCRCC 26 years ago; this patient visited our hospital with abdominal pain due to multiple abdominal metastases 24 years after the nephrectomy. After metastasectomy, he underwent targeted therapy combined with a programmed death receptor-1 (PD-1) inhibitor, and the current imaging results indicate remarkable tumor remission.

**Conclusions:**

Metachronous pancreatic metastasis from CCRCC after nephrectomy is rare, but clinicians and patients should not ignore this possibility. The combination of targeted therapy and immunotherapy can result in satisfactory outcomes in cases where metastatic CCRCC continues to progress despite metastasectomy and targeted therapy. The combination of local and systemic therapy can be an effective therapeutic protocol for metastatic CCRCC, but there is no consensus on suitable therapeutics.

## Introduction

Renal cell carcinoma (RCC) is a malignant tumor originating from the renal epithelial cells and accounts for 80%–90% of all kidney malignancies (1). Approximately 25% of all patients with RCC have distant metastases at diagnosis, and more than one-third of all patients will develop metastases after nephrectomy (2). Clear cell renal cell carcinoma (CCRCC) is the most common histopathological type of RCC, and it originates from the proximal tubular epithelial cells that can invade the renal sinus and extend to the renal vein (3). Consequently, vascular invasion by CCRCC is more common than other histopathological types of RCC. Compared to metastases to the liver, lung, adrenal gland, bone, or brain, RCC metastases to the pancreas are rare and account for 1%–4% of all malignant pancreatic tumors (4, 5). Clinically, imaging modalities that can effectively differentiate metastases from primary pancreatic tumors include enhanced computed tomography (CT), positron emission tomography (PET) associated with CT (PET/CT), magnetic resonance imaging, and endoscopic ultrasound-guided fine-needle aspiration biopsy (3). Here, we describe a case of CCRCC metastases to the pancreas and other distant organs 24 years after radical nephrectomy and review the relevant literature.

## Case Report

**Figure 1:**
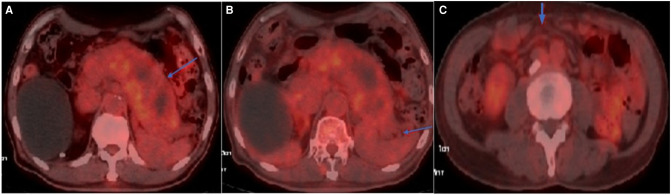
Axial view of FDG-PET/CT: (**A**) Hypermetabolic activity in the swollen pancreas with SUV_max_ of 3.0; (**B**) Mild hypermetabolic activity surrounding the pancreas; (**C**) Mild hypermetabolic activity in the mesenteric area with SUV_max_ of 1.9.

**Figure 2:**
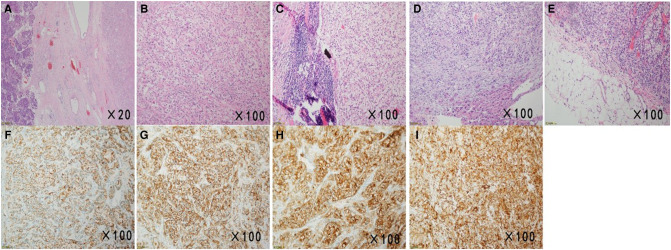
Micrographs of the surgical specimens and immunohistochemical examination results. (**A,B**) Sections of the pancreas showed atypical clear cells monomorphonuclear with bright cytoplasm (**H&E**, ×20, ×100). (**C–E**) HE staining image of the peripancreatic *lymph* node, sections of the liver, and the omental *tissue* showed histological features similar to those of the pancreatic sections (**H&E**, ×100, ×100, ×100). (**F–I**) Immunohistochemical examination of surgical specimens showed neoplastic cells with CK (+), CA9 (+), CD10(+), and Vimentin (+) (×100).

A 76-year-old Asian man underwent radical left nephrectomy due to CCRCC in 1995, but detailed medical records of the primary renal tumor were not available as the patient had undergone the procedure at another hospital. In August 2019, the patient suddenly experienced epigastric pain after eating, accompanied by abdominal distension, nausea, and vomiting. Enhanced abdominal CT revealed a space-occupying lesion in the pancreatic body and tail, multiple liver metastases, and lymph node metastases around the pancreas; however, chest CT was negative. CT imaging findings of the space-occupying lesions were similar to those of CCRCC. Whole-body fluorodeoxyglucose PET/CT scans showed pancreatic thickening along with increased fluorodeoxyglucose tracer intake, pancreatic duct dilation, and a mild increase in the metabolic activity of lymph nodes in the peripancreatic and mesenteric areas (Figure 1). Thus, as the patient’s symptoms, past history, and imaging results were indicative of malignant tumor metastasis, we did not perform a biopsy but chose to proceed with metastasectomy. The patient underwent combined exploratory laparotomy with pancreatic body and tail resection, splenectomy, and left hepatic lobectomy. In the early stage of surgery, the intraoperative frozen-section examination was performed to identify the tumor property. The dimensions of the excised pancreatic tumor were 15 × 7 × 6 cm, and the cut surface was multicolored. Metastases were visible in 1 of 3 lymph nodes around the pancreas, whereas the two liver metastases measured 2 × 1 × 3 cm and 1.5 × 1 × 2 cm, respectively. The results of histopathological and immunohistochemical analyses are provided in Figure 2. Histologically, the CCRCC metastases were found in Fuhrman grades II–III. Considering the adverse reactions and financial burden of the systemic therapy, we decided to follow up closely without the application of systemic therapies for the time being.

**Figure 3:**
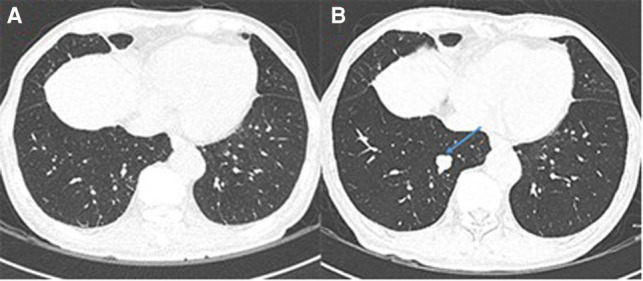
Newly-occurred lung metastases after metastasectomy. (**A**) No lung metastases before metastasectomy. (**B**) The metastatic lesion in the right lower lobe (arrow) 10 months after metastasectomy.

**Figure 4:**
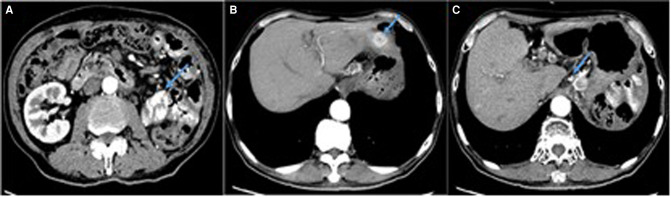
Enhanced CT scan showing multiple abnormal enhancement lesions (arrows). (**A**) The mass with heterogeneous enhancement in left kidney area. (**B**) A ‘target-like’ sign and heterogeneous enhancement of the nodule in liver. (**C**) Multiple enlarged lymph nodes in hepatic hilar region.

In June 2020, the high-resolution chest CT re-examination revealed new nodules in the basal segment of the right lower lobe (Figure 3). We believed that systemic therapy was necessary to prevent tumor progression and prolong survival. Then the patient received targeted therapy (Sunitinib). At this time point, the patient had a Memorial Sloan Kettering Cancer Center (MSKCC) (6) intermediate-risk feature based on clinical manifestations and laboratory examinations. In September 2020, a reexamination of the abdominal enhanced CT showed that there were multiple new-onset metastatic lesions in the left renal area, the liver, and the lymph nodes in the hilar area (Figure 4). Given the continuous progress of the tumor, we decided to use targeted therapy combined with the PD-1 inhibitor (Toripalimab). In May 2021, a chest CT scan showed no significant changes in pulmonary nodules*;* while an abdominal CT scan revealed that the number of liver metastases was reduced and the size was decreased. Consequently, we could conclude that the patient had a partial response after receiving targeted therapy combined with PD-1 inhibitors. Since receiving the combinatorial treatment, the patient has not suffered from any serious adverse events. Conversely, the patient’s physical and psychological conditions are gradually improving. We will continue to adopt targeted therapy combined with immunotherapy, and follow up regularly.

## Discussion

CCRCC is the most common and aggressive type of RCC. At the time of diagnosis, the metastasis has usually occurred due to the asymptomatic features of CCRCC, and more than 20% of CCRCC patients relapse after nephrectomy (7). CCRCC can metastasize to almost all organs or tissues. The earliest and most common metastatic site is the lung, followed by the liver, adrenal glands, bones, brain, and pancreas. Pancreatic metastases account for only 2%–5% of pancreatic malignancies (8). Among pancreatic metastases, CCRCC metastasized to the pancreas accounted for only 1.3% (9). Even though mechanisms underlying metachronous CCRCC metastases after radical nephrectomy have not been elucidated, we hypothesize that this patient must have been chronically harboring CCRCC micrometastases and immunosurveillance served to maintain a relative balance between micrometastases and the immune system. Arguably, this equilibrium lasted 24 years but was disrupted by an event/events that led to tumor progression, and we propose two potential reasons as follows. First, the tumor gradually established a stable tumor microenvironment (TME) at the metastatic site, which then promoted angiogenesis and induced immune tolerance by releasing cytokines (10). Second, physiological changes due to aging during 24 years, such as inflammaging and immunosenescence (11, 12), could have triggered progression. Inflammaging is defined as a chronic state of low-grade inflammation characterized by elevated levels of inflammatory mediators and proinflammatory cytokines and is attributed to long-term endogenous or exogenous factors. However, to avoid excessive inflammatory reactions, the immunosuppressive network is activated, which not only increases the number of immunosuppressive cells and secretion of anti-inflammatory cytokines but also decreases positive immune function(s). These long-term changes in the immune system result in immunosenescence (11). Additionally, long-term stimulation of micrometastases could have contributed to inflammaging and immunosenescence, thereby resulting in a decline in immunosurveillance, which then weakened the limitations on tumor growth and metastasis. Thus, we suggest that the formation of a conducive TME and immunosenescence together contributed to the failure of immunosurveillance.

Although the detection of metastases usually takes a long time from the diagnosis of primary renal tumors, for example, the average time of pancreatic metastasis is 7.1–10.0 years (13), metastasis occurred 24 years after radical nephrectomy in the present case. To the best of our knowledge, this situation is rare, and to date, the longest duration from diagnosis to metastasis in CCRCC is 32.7 years (13). Hence, we propose that clinicians design patient-centered follow-up plans based on the primary tumor to ensure an optimal cost-benefit ratio.

At present, there is still a lack of effective methods for the examination of metastatic CCRCC. Imaging examination is the main diagnostic method. However, the imaging manifestations of most metastatic tumors are similar to those of tumors originating from the metastatic site, which might lead to misdiagnosis. Notably, when the tumor metastasizes to a single organ or site. The preoperative misdiagnosis rate reaches about 70% (14). The examination measures commonly used for the metastatic tumors of the pancreas or liver include enhanced CT, enhanced MRI, PET/CT, PET/MRI, and endoscopic ultrasound-guided biopsy (3, 15, 16).

For metastatic CCRCC, there are currently two treatment options, including local therapies and systemic therapies. Local therapies include cytoreductive nephrectomy, metastasectomy, and stereotactic radiotherapy (SRT) (17–19). Systemic therapies include targeted therapy and immunotherapy (20, 21). It is generally believed that local therapies should be used in combination with systemic therapies. Yet, it has also been shown in several studies that whether combined with local therapies had little influence on the effects of systemic therapies (22). Local therapies can even increase risks, such as surgical trauma, iatrogenic metastasis, and radiotherapy toxicity.

It is believed that cytoreductive nephrectomy and metastasectomy can reduce tumor growth factors, inflammatory cytokines, and immunosuppressive factors secreted by cancer cells, thereby reducing tumor growth or improving the effects of systemic treatment (23). Furthermore, the survival and recurrence-free interval after pancreatic metastasis resection of CCRCC is significantly longer than in other tumors according to a previous meta-analysis of pancreatic metastasis of malignant tumors (24). However, the incidence of surgical complications and mortality is relatively high for metastatic RCC patients 75 years or older (25). Consequently, clinicians need to be cautious in choosing whether to perform surgery. Given the existing literature, we believe that whether to perform cytoreductive nephrectomy or metastasectomy for patients with metastatic CCRCC cannot be generalized. The final choice should be based on a comprehensive assessment of clinical manifestations, degree of frailty, pathological type, metastatic location and burden, drug treatment response, and life expectancy.

SRT is an emerging treatment method for metastatic CCRCC. Although CCRCC was considered to be radiation resistant in the past, SRT can provide very high local doses to kill tumor cells (22). More importantly, several prospective trials have exhibited that radiotherapy might induce PD-1 ligand 1(PD-L1) expression in tumor tissues, thereby increasing the anti-tumor ability of immune checkpoint inhibitors (ICIs) (26). A retrospective study by Kroeze *et al*. (27) showed that targeted therapy or immunotherapy combined with SRT could significantly improve the progression-free survival and overall survival of metastatic RCC patients without increasing toxic effects. As far as we know, SRT has been applied to metastases of RCC to lungs, bones, lymph nodes, liver, intracranial, and spinal (22).

Recently, the systemic therapies of metastatic CCRCC have undergone tremendous changes. The use of vascular endothelial growth factor (VEGF) inhibitors alone is no longer recommended. The combination of PD-L1 inhibitors and cytotoxic T-lymphocyte-associated antigen 4 (CTLA-4) inhibitors or PD-L1 inhibitors and VEGF inhibitors is currently recommended (28). ICIs and VEGF inhibitors are both first-line standard treatments for metastatic CCRCC (7). The VEGF targeted therapy can enhance the infiltration capacity of T cells, which provides a strong theoretical basis for the combination of VEGF targeted therapy and ICIs therapy in metastatic CCRCC (29). Additionally, Li *et al*. confirmed that Sunitinib could inhibit the PD-L1 expression in tumor tissues (30), which furnishes another supporting evidence for Sunitinib combined with ICIs. The combination of immunotherapy and targeted therapies has shown potent metastases suppressing effects in both first-line and second-line treatment, and prolonged the overall survival (29).

At present, there is still controversy about the specific regimen of the combination of systemic therapies and local therapies. Most researchers consider that local therapies should be used after systemic therapies (31, 32). On the one hand, great progress has been made in systemic therapies. Some metastatic CCRCC patients can achieve desired outcomes with systemic therapies, and there is no significant difference in whether combined with surgical treatment (33). In the case that systemic therapies effectively control disease progression, patients can avoid trauma or related complications caused by local therapies. On the other hand, local therapies are able to address the puzzle of drug resistance in tumors after systemic therapies. However, some researchers believe that local therapies will provide a certain drug-free interval, thereby reducing adverse events caused by systemic therapies and maintaining the quality of life (34, 35). Therefore, local therapies should precede system therapies.

This patient had a partial response after receiving targeted therapy combined with PD-1 inhibitors. Thus, the inhibitory effects of Sunitinib and Toripalimab on metastatic CCRCC are obvious and encouraging. During the combinatorial treatment, this patient did not experience serious adverse reactions or complications. However, Liu *et al*. found that ICIs combined with radiotherapy promoted the development of lung metastases from CCRCC (36). This might be because ICIs significantly activated and increased tumor-infiltrating PD-1^+^ Treg cells and led to suppression of anti-tumor immunity (37). Therefore, we should review the changes in patients with metastatic CCRCC regularly when applying ICIs, such as PD-1 inhibitors.

In the case of multiple organ metastases from CCRCC, the European strategy is more inclined to initiate a systemic treatment first. Next, whether a local treatment will be proposed depends on the response to systemic treatment (38). Nevertheless, our study suggests that systemic therapy following metastasectomy may be also safe and effective. Certainly, the final clinical decision should be made after a comprehensive analysis of the patients’ situation, which is in the best interest of the patients.

## Conclusions

Although metachronous pancreatic metastasis of CCRCC after radical nephrectomy is rare, clinicians and patients should not ignore this possibility. The combination of targeted therapy and immunotherapy can result in satisfactory outcomes in cases where metastatic CCRCC continues to progress despite metastasectomy and targeted therapy. However, the effectiveness and safety of such a treatment scheme require large-scale research to verify in the future. The combination of local and systemic therapy can be an effective therapeutic protocol for metastatic CCRCC, but there is no consensus on suitable therapeutics.

## Data Availability

The original contributions presented in the study are included in the article/Supplementary Material, further inquiries can be directed to the corresponding author/s.
